# Design and commissioning of a multi-mode prototype for thermochemical conversion of human faeces

**DOI:** 10.1016/j.enconman.2018.02.065

**Published:** 2018-05-01

**Authors:** Nelia Jurado, Tosin Somorin, Athanasios J. Kolios, Stuart Wagland, Kumar Patchigolla, Beatriz Fidalgo, Alison Parker, Ewan McAdam, Leon Williams, Sean Tyrrel

**Affiliations:** School of Water, Energy and Environment, Cranfield University, Cranfield, Bedfordshire MK43 0AL, United Kingdom

**Keywords:** Combustion, Smouldering, Human faeces, Micro-combustor, Updraft, Downdraft, Nano-membrane toilet, Design

## Abstract

This article describes the design and commissioning of a micro-combustor for energy recovery from human faeces, which can operate both in updraft and downdraft modes. Energy recovery from faecal matter via thermochemical conversion has recently been identified as a feasible solution for sanitation problems in low income countries and locations of high income countries where access to sewage infrastructures is difficult or not possible. This technology can be applied to waterless toilets with the additional outcome of generating heat and power that can be used to pre-treat the faeces before their combustion and to ensure that the entire system is self-sustaining. The work presented here is framed within the Nano Membrane Toilet (NMT) project that is being carried out at Cranfield University, as part of the Reinvent the Toilet Challenge of the Bill and Melinda Gates Foundation.

For this study, preliminary trials using simulant faeces pellets were first carried out to find out the optimum values for the main operating variables at the scale required by the process, i.e. a fuel flowrate between 0.4 and 1.2 g/min of dry faeces. Parameters such as ignition temperature, residence time, and maximum temperature reached, were determined and used for the final design of the bench-scale combustor prototype. The prototype was successfully commissioned and the first experimental results, using real human faeces, are discussed in the paper.

## Highlights

• Ignition, gasification and combustion of simulant and real faeces were studied.
• Trials using fuel flowrates of 1.2 g/min and 7.5–8 L/min of air were carried out.
• Mean temperatures of 440–670 °C allowed self-sustained combustion.
• Maximum temperatures reached for real faeces were in the range of 1210–1240 °C.
• Combustion trials lasted up to 160 min without external heat supply.

## 1 Introduction

The development of alternative sources of energy and the need to protect the environment is changing our perspectives on the way natural resources are utilised and waste is managed. Materials such as municipal solid waste, agricultural residues and sewage sludge that were traditionally considered as waste, and either burnt or discarded into landfills, are now regarded as feedstocks for bioenergy [1], [2], [3], [4]. Human faeces are one of such biomass resources with potential to be used as fuel and converted to heat and/or electricity [5].

The ‘Reinvent the Toilet Challenge’ funded by the Bill & Melinda Gates Foundation was launched to improve access to affordable, safe and sustainable sanitation while simultaneously utilising the chemical energy contained in human faeces. The challenge aims to develop innovative household-scale toilets that treat human excreta and recover useful resources at affordable price without producing hazardous products [6], [7], [8], [9]. The successful implementation of reinvented toilets for human waste implies a cultural shift in the use of natural resources and a significant contribution to reduce environmental problems associated with non-optimal ways of disposing of faeces in developing countries, e.g. open defecation into the environment without treatment [10]. It is expected that reinvented toilets will have an impact on the re-design of conventional sanitary systems and encourage the efficient use of energy. This article discusses and presents the concept of human waste-to-energy being exploited within the development of the Nano Membrane Toilet (NMT). NMT is a domestic-scale sanitary solution intended for people with limited access to sanitation, water and electricity infrastructures. The NMT is under development at Cranfield University. It aims to operate with a flush mechanism that avoids the use of water, and to achieve self-sustained operation by valorising the faeces without the need for external energy supply. Moreover, any residual energy obtained from the faecal matter, which is not used for the NMT operation, could be used to power small domestic appliances such as mobile phones. The thermodynamic analysis recently conducted by our group [11] has proved the possibility of the self-sustained operation of the NMT and the need for the efficient management of the energy recovered from the human faeces in order to generate sufficient power for the auxiliary equipment.

Onabanjo et al. [5] and Muspratt et al. [12] showed that human faeces have a comparable and, in some instances, higher heat value than wood biomass (both on a dry basis). This chemical energy can be recovered via thermochemical conversion technologies including smouldering [13], [14], combustion [5], [15], hydrothermal carbonization [10], [16], and pyrolysis [17]. Combustion presents a huge opportunity for energy recovery in the NMT because of the heat released during the process at temperatures ranging from as low as 250 °C (smouldering) to >1000 °C (combustion), depending on air excess, ignition modes (standard vs. booster), fuel composition, etc. The released heat is required for removing moisture from fresh human faeces prior to being fed into the micro-combustor [5]. Contrary to other thermochemical processes such as pyrolysis and hydrothermal treatment, combustion is a mature and widely applied process, thereby increasing its potential application for NMT and similar sanitary solutions where affordability is a priority, particularly for developing countries. For a domestic-scale toilet, a continuous mode of operation of the micro-combustor would be desirable to limit the energy requirement for ignition and to ensure that the bed material is sufficiently hot for incoming partly-moist faecal material.

Work from our group using a bench-scale reactor [18] showed the feasibility of combustion of faeces; however, the need for a controlled air supply for self-sustained ignition and flame propagation was emphasised. The paper resulting from that work also highlighted the need for removing the ash, which is the reason for proposing a regulated ash removal system, in the present work, that can prevent the build-up of ash in the combustion zone but in a sufficient amount to still retain heat for continuous thermal treatment. To ensure the safety of users and minimise heat loss, which increases thermal efficiency, special attention was paid to material selection for the micro-combustor. In addition, it was concluded that the capacity of the bench-scale reactor (in the range of 1.5–2.3 g/min) was too large for an NMT designed for a single household with a ten-user capacity, and lower faeces burn rate of about 0.4 g/min is required [5]. A flexible mode of operation is also desirable to accommodate different toilet user capacities, faeces generation rates, and periods of absence of the users.

The justification for designing a new prototype for the combustion of human faeces in the context of the NMT project was based on results from previous experimental studies carried out with a bench-scale facility [18], designed at RTI International/Colorado State University and tested at Cranfield University. This new design was needed for a smaller community (which will affect the scale of the system) and will look at a continuous process, in contrast to the batch operation performed with the previous bench-scale rig. Additionally, the prototype of the micro-combustor needs to allow for flexibility of operation, so it can be used to evaluate different operating modes and assess the most favourable operating parameters for the NMT. Thus, the focus of this study is to build and commission a micro-combustor system that is suitable for the thermochemical conversion of human faeces and the continuous, self-sustained operation of the NMT. The strategy adopted for this study was to develop the prototype in two stages. In the first stage, preliminary trials were carried out to characterise the main operating parameters of the system (explained in detail in Section 2.1); and, the second stage was dedicated to the design of the prototype using the information obtained during the first stage and generating more experimental data to adapt and enhance the operability of the final prototype.

## 2 Design of the prototype

The procedure followed to design the micro-combustor consisted of two stages: preliminary experimental study to understand the key parameters that govern the thermochemical conversion of human faeces at this process’ scale, and the design of the prototype.

During the preliminary stage the operating parameters included:

– Heating rate of the fuel to ignition;
– Ignition temperature;
– Combustion time and temperature;
– Characteristics of the solid residue.

The collected information was used to size the reactor for an NMT for a household of ten people. Additionally this reactor must ensure continuous operation. Two important requirements drove the design:

1. Minimisation of the heat loss within the combustion chamber. Due to the scale’s very low fuel flowrate, it is crucial to avoid heat loss by the adequate selection of the material for the combustion chamber and appropriate sizing of the insulation material.
2. Flexibility of operation. The prototype unit is able to operate both as updraft and downdraft with the objective of establishing the most adequate operating mode and increasing the knowledge about the thermal conversion of human faeces on a small scale, which is currently very limited [5].

### 2.1 Preliminary experimental study

#### 2.1.1 Fuel characterisation

Wood pellets, simulant faeces pellets and human faeces were tested as fuels in the experimental trials. The wood pellets were produced at Cranfield University. The simulant faeces pellets were produced following the recipe showed in Table 1 [19] and dried for 24 h in an oven at 45 °C. The human faeces were collected and characterised under the approved procedures of the Cranfield University Research Ethics Scheme. The detailed description of the methods followed to obtain the elemental analysis of the human faeces tested, the moisture content of the samples used, and the calculation of the heat value for the simulant faeces and human faeces, can be found elsewhere [5]. The proximate and ultimate composition, particle size, bulk density, and calorific value of the fuels used are presented in Table 2.

**Table 1:** Recipe for simulant faeces [19].

Ingredients	Dry weight (g/kg)
Baker’s yeast	72.8
Peanut oil	38.8
Miso paste	24.3
Propylene glycol	24.3
Cellulose powder	12.4
Psyllium husk powder	24.3
Calcium phosphate	25
Water*	778.1

* Water added based on the required moisture content.

**Table 2:** Chemical and physical properties of the types of biomass used as fuel.

Biomass type	Bulk density (kg/m^3^)	Part. av. size (mm)	Proximate analysis (wt.% db)			Ultimate analysis (wt.% db)				Moisture content (wt.% wb)	HHV (MJ/kg)
			VM	FC	Ash	C	H	N	O^*^		
WB	600 ± 18	8 × 10	98.70	0.22	1.04	48.96	6.88	0.08	43.04	–	21.54
SF	626 ± 5	4 × 10	82.00	0.00	18.00	45.51	6.63	3.34	26.51	77.00	21.53
HF	277 ± 45	5 × 7	82.00	0.00	18.00	48.15	6.92	5.29	21.64	79.00	22.03

WB: Wood Biomass pellets; SF: Simulant Faeces; HF: Human Faeces; (^*^): 100 – (wt. % of C, H, N and ash); db: dry basis; wb: wet basis; VM: Volatile matter; FC: Fixed carbon; HHV: High heating value.

#### 2.1.2 Preliminary experiments

**Fig. 1:**
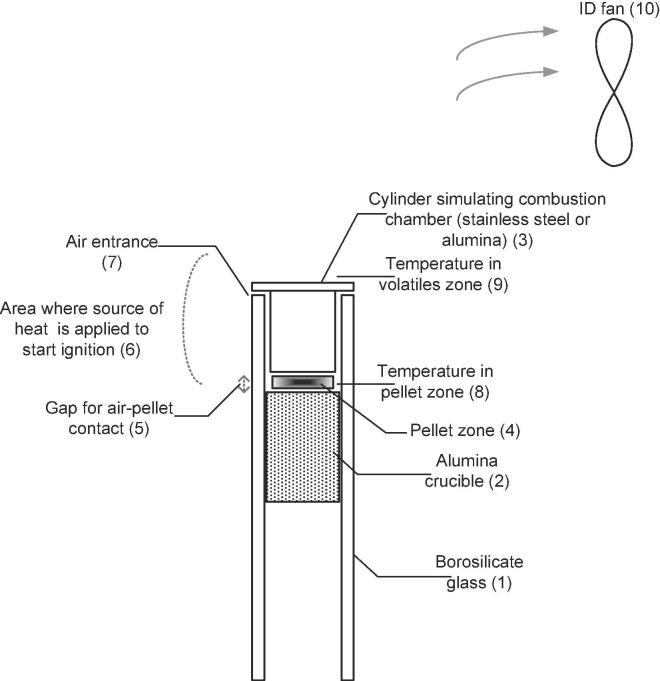
Schematic of the experimental set-up to simulate 24 h/day operation of the combustor (0.43 g/min).

**Fig. 2:**
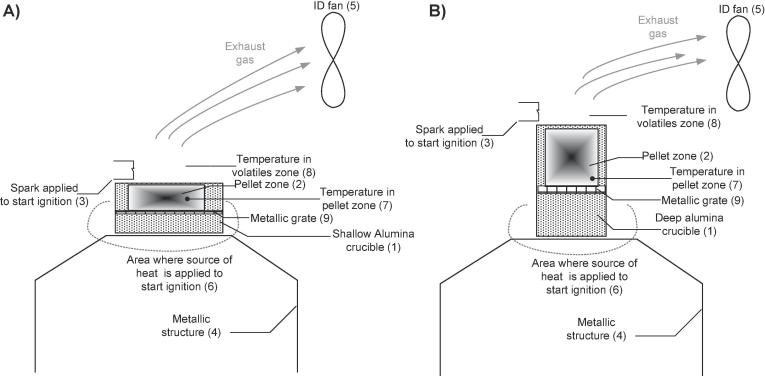
Schematic of the experimental set-up to simulate 8 h/day operation of the combustor (1.2 g/min). (A) Shallow crucible; (B) deep crucible.

Batch experiments were performed using wood pellets and simulant faeces pellets as fuel, and carried out in the experimental set-ups shown in Fig. 1, Fig. 2.

The experiments were performed to replicate a fuel flowrate of 625 g of faeces (db) per day as required for the scale of the prototype. This value is calculated by considering 25 wt.%db faecal matter for ten users at average faecal generation rate of 250 g/cap/day, which results in a total of 2.5 l of excreta per day. Two operating periods were considered: 24 h/day, which requires a flowrate of dry faeces of 0.43 g/min; and 8 h/day which requires a flowrate of dry faeces of 1.2 g/min.

In the case of 24 h/day operation, various configurations were studied to allow for longer residence times and higher bed temperatures. Alumina and stainless steel combustion chambers (Ø18 mm × H27 mm) were tested. Three heights of annular gaps for entry of air, small (2 mm), medium (4 mm) and large (6 mm), were used (5) in Fig. 1. In addition, both natural and induced convection, using an induced draft fan ((10) in Fig. 1 and (5) in Fig. 2), were considered. In the case of 8 h/day operation, two combustion chambers, shallow (Ø62 mm × 18 mm (height)) and deep (Ø49 mm × 80 mm (height)) were used (see Fig. 2). Both crucibles were made of alumina in order to minimise the heat loss and allow for longer combustion times.

In the case of 24 h/day operation of the combustor, the crucibles were heated by a blow torch located around the area denoted as (6) in Fig. 1. Heating stage 1 (noted as ‘t1’) was considered until volatiles started to release (visible as a white smoke). After the heating stage, the blow torch was directed to the freeboard zone ((9) in Fig. 1). Heating stage 2 (recorded as ‘t2’) lasted until ignition occurred. Combustion stage (noted as ‘t3’) was considered while the flame was visible and until its extinction.

Similar to 24 h/day operation, the crucibles were slowly and uniformly heated with a blow torch in the case of 8 h/day operation of the combustor. Heating stage 1 was considered until the temperatures in the pellet zone reached 150 °C. Ignition was then forced with a spark in the freeboard zone. The ignition temperature was different depending on the configuration and the spark was applied every 10 °C-interval until ignition occurred (e.g. spark applied at 150 °C, 160 °C, 170 °C, 180 °C, etc.). Tests were considered null if the pellet zone reached 300 °C without having achieved ignition of the fuel. Complete release of the volatiles was considered at this temperature, with the consequent inability to ignite the solid residue using the sparking device. Similarly to the 24 h/day operation’s tests, times ‘t1’, ‘t2’, and ‘total combustion time’ were noted during these trials. For the experiments where batch feeding was tested, the pellet(s) were fed into the crucible at a rate of 0.43 g every 20 s, and the total number of batches was noted. This way of supplying the fuel was selected because it was observed to work better during the initial tests: feeding the pellets in little batches but more frequently created a more sustained combustion than feeding a 1.2 g sample every minute, as the latter usually suffocated the flame.

#### 2.1.3 Preliminary results

The experimental trials carried out during the preliminary phase of this work, generated data regarding temperatures measured at different locations; heating-up, ignition and complete combustion times were also recorded. These data were used as reference for the design of the micro-combustor prototype.

**Fig. 3:**
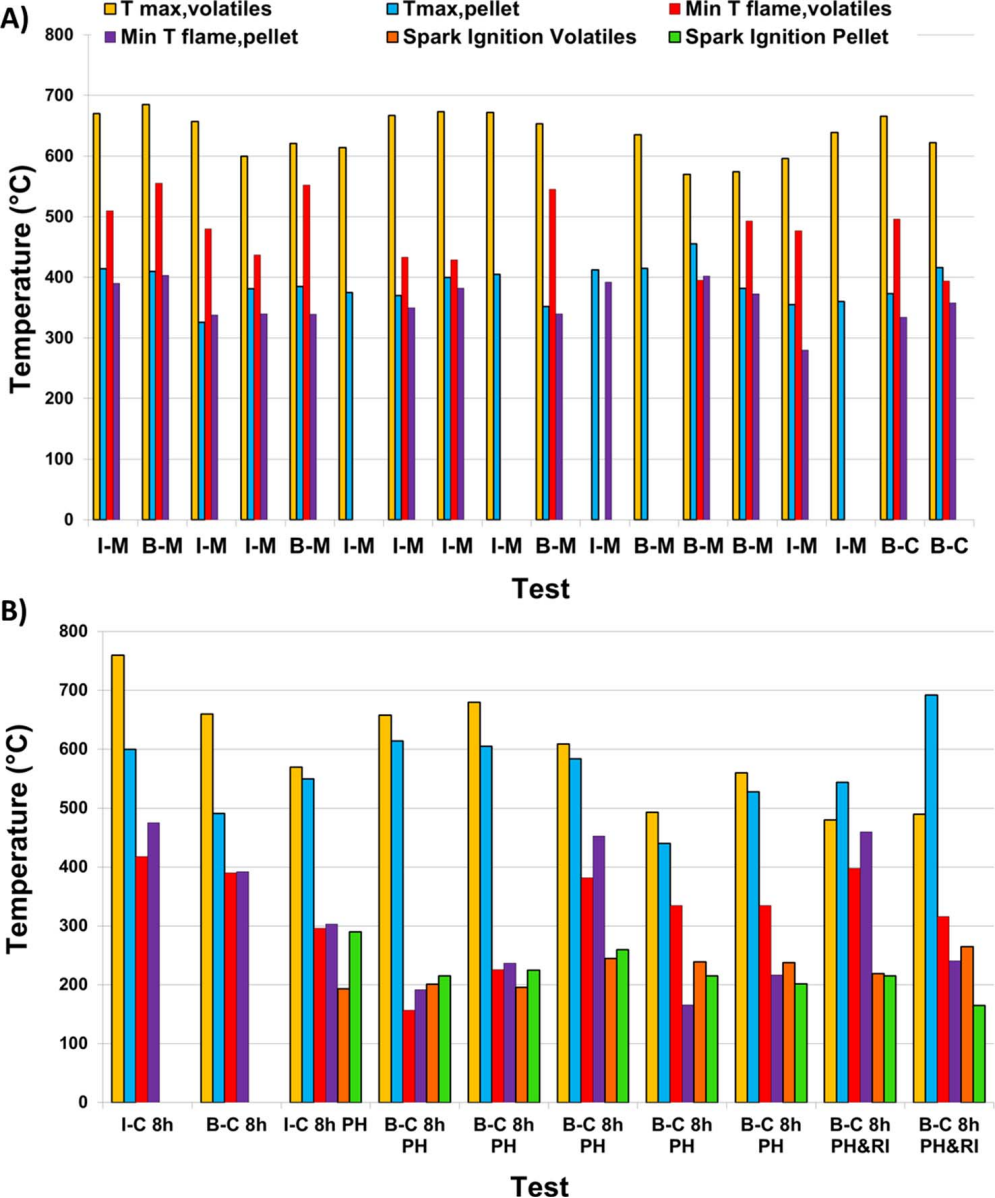
Experimental results for preliminary tests using: (A) 24 h/day operation set-up; (B) 8 h/day operation set-up.

Fig. 3 shows the main temperatures observed during some of these preliminary tests; only data for tests using simulant faeces pellets have been included here as they were the most significant ones to consider for the design of the prototype. The values presented in these plots are explained in Table 3.

**Table 3:** Main temperatures logged during preliminary tests and the location where measured.

Parameter	Description	Notes
T_max, volatiles_	Maximum temperature in the zone of the volatiles	In (9) in Fig. 1
T_max, pellet_	Maximum temperature in the zone nearby the pellet	In (8) in Fig. 1
Min T_flame, volatiles_	Minimum temperature in the zone of the volatiles	In (9) in Fig. 1 In the instant just before the flame extinguished
Min T_flame, pellet_	Minimum temperature in the zone nearby the pellet	In (8) in Fig. 1 In the instant just before the flame extinguished
Spark ignition volatiles	Temperature measured at the volatiles zone	In (9) in Fig. 1 At the ignition of the pellet; i.e. flame visible
Spark ignition pellet	Temperature measured nearby the pellet	In (8) in Fig. 1 At the ignition of the pellet; i.e. flame visible

The preliminary tests shown in Fig. 3 were named as A-B-C-D where the meaning of each parameter are described in Table 4.

**Table 4:** Nomenclature used for preliminary tests.

Order/meaning	Identifier	Specific cases
A : simulant faeces pellet feeding	I: individual pellet	
	B: batch pellet feeding at the rate defined by the type of test simulated	0.43 g/min for 24 h/day operation, or 1.2 g/min for 8 h/day operation
B: material of the combustion chamber (cylindrical body)	M: metallic (stainless steel)	Location (3) in Fig. 1
	C: ceramic (alumina)	Location (3) in Fig. 1
C:operating period simulated	8 h	Fuel_feeding rate_: 1.2 g/min
	24 h	Fuel_feeding rate_: 0.43 g/min
D: other features during tests	PH: crucible preheated	T_crucible_: 100–110 °C prior to feeding the first pellet
	RI: spark applied to re-start the combustion	During the test

Reignition of the pellets by using a low-voltage sparking device was only possible if the spark was applied when temperature of the extinguished combustion bed was higher than 165 °C.

The maximum temperatures reached in the volatiles zone for the 24 h/day operation set-up are in the range between 550 °C and 750 °C; however, that range is wider, reaching lower values, between 480 °C and 760 °C, for some cases using the 8 h/day set-up. This is believed to be a consequence of the heat generated by the combustion being kept in the pellets bed instead of being released to the volatiles zone. The results obtained for the temperatures in the pellet zone during the last five tests using the 8 h/day operation set-up (i.e. right hand-side of Fig. 3B: B-C 8h PH; B-C 8h PH; B-C 8h PH; B-C 8h PH&RI; B-C 8h PH&RI) corroborate this fact: temperatures achieved in the pellet zone (440–690 °C) were higher than the temperatures measured in the volatile zone. In the experiments with T_volatiles zone_ higher than T_pellet zone_, the differences observed between both temperatures was lower for the 8 h/day operation set-up than for the 24 h/day operation set-up. The minimum temperatures measured in the volatile zone for the tests using 24 h/day operation set-up range between 430 °C and 550 °C, which are higher than those measured for the 8 h/day operation set-up when the flame subsisted between 150 °C and 420 °C. The same observation can be made when considering the minimum temperatures measured in the pellet zone before the flame extinguished: the tests undertaken using the 8 h/day operation set-up show a wider range of temperatures from 175 °C to 475 °C, than those with the 24 h/day set-up which range from 290 °C to 405 °C. It can be extracted from these remarks that the pellets bed acts as a heat buffer, making the maximum temperatures reached in the volatiles zone lower, keeping the heat in the pellet zone, which means that the flame can survive at lower temperatures. This was an important input to the prototype design. Namely, allowing for the accumulation of the fuel pellets is needed, and hence to plan the necessary volume in the reactor for that build-up, so the combustion or smouldering can continue over a wider range of temperatures.

For the study of the ignition characteristics, two different procedures were applied: using a blow torch with the 24 h/day set-up (i.e. Fig. 3A), and applying a spark, once the fuel had been preheated, for the tests with 8 h/day set-up (i.e. Fig. 3B). The temperatures measured at the ignition instant were very similar between the volatiles and pellet zone, keeping within the range of 165–290 °C.

The ranges of times observed empirically for t1, t2 and t3, were used to establish the residence time that a certain amount of fuel needed to achieve a high level of combustion or smouldering, maximising, in this way, the levels of burnout achieved in the ashes generated. The values for the times noted are within the ranges presented below:

– t1: 30–50 s for 24 h/day operation tests; and 99–148 s for 8 h/day operation tests;
– t2: 70–82 s for 24 h/day operation tests; and 210 s for 8 h/day operation tests;
– Combustion time, t3: 70 s for 24 h/day operation tests; and 100 s for 8 h/day operation tests.

Additionally a number of interesting facts were noted during the trials carried out with the 24 h/day operation set-up:

– Smaller batches of pellets fed more frequently kept the flame alive for longer;
– The volatiles were visible earlier (t1 observed around 20–30 s) for the tests done with the ceramic crucible located at a lower position;
– The tests performed with the ceramic crucible located at a higher position presented longer combustion times although the appearance of the flame was delayed;
– Longer combustion times were achieved when using the ceramic crucible in comparison with the cylindrical metallic body;
– The smouldering of the pellets was observed and noted to take place up to 2 min after the flame had extinguished.

The empirical facts, observed during the preliminary tests, suggested considering the following aspects for the operation of the bench-scale prototype:

– Procedure for starting ignition. The option that appeared to work best was to preheat the pellet zone up to 150–200 °C, i.e. the temperature at which the highest release of volatiles from the fuel is produced. A spark ignition must then be applied at the volatile zone to start the ignition of the fuel.
– Zoning the combustion chamber. In some cases, a high percentage of unburnt pellets was observed, which indicated the need for longer residence times. The solution implemented in the design of the prototype to avoid or minimise the unburnt content in the ashes, is to create various zones in the combustion chamber: warming-up, combustion, and char-to-ash zone. These zones are shown in the subsequent section, dedicated to the description of the prototype.

### 2.2 Design of the micro-combustor prototype

*Modes of operation.* Two modes of operation were considered for the micro-combustor prototype: updraft and downdraft. Each of them has positive and negative aspects regarding operational issues (e.g. tars generation). The flexible design of the prototype developed for this work will enable a good understanding of the preferred operating mode to fulfil the requirements from the NMT.

*Materials selection.* The combustion chamber should be made of a material that minimises the heat loss (e.g. ceramic – more specifically alumina) to ensure a sustained combustion. However, it must also be made of a material that allows for the flexibility of applying different configurations using the same prototype; in other words, easily machinable (e.g. metal, more specifically stainless steel). The necessity of having a prototype with dual characteristics led to the consideration of a modular design where most of the body of the combustion chamber would be made of alumina, and using stainless steel in those sections that it would be necessary to machine to add inlet/outlet ports. Also, by interchanging certain modules the updraft and downdraft configurations could be tested. The main issue this alternative presented was the prototype could crack easily in the adjoining points between the modular sections due to the different expansion behaviour between stainless steel and alumina. Therefore, another material for the metallic sections was considered, such that it would have similar characteristics towards thermal expansion as alumina. A search to find a compatible material was carried out and it was concluded that Kovar alloy [20] responded to this requirement.

Two main techniques were found to be available for the union between the Kovar alloy and the ceramic material: diffusion bonding [21] and active brazing [22] (using a third material interlayer). The active brazing requires a definitive union.

Acknowledging the fact that the modular design was not feasible at this stage, it was decided that a ceramic material should be used for the whole body of the combustion chamber. This ceramic material should respond to two important requirements: ability to be modified by mechanical means, and to have a similar thermal expansion to stainless steel. The latter is due to the connections that this unit must have with the inlet/outlet ports which will be made of stainless steel. The search was successful and a glass-ceramic material called Macor™ [23] was found, which responded to the requirements needed for our application.

**Fig. 4:**
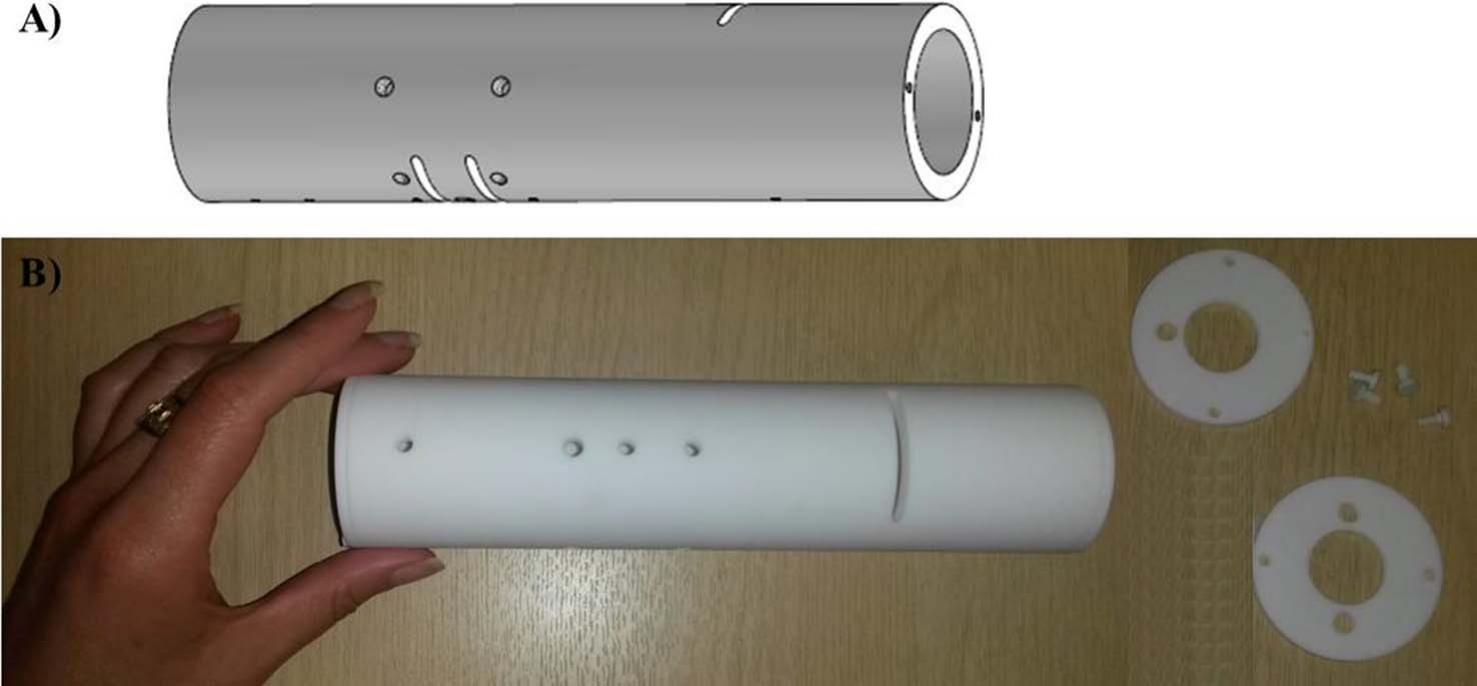
Combustion chamber manufactured in glass-ceramic material: (A) Plot used for design; (B) actual piece made of Macor™ with lids and caps.

*Final set-up.* The design of the prototype for the thermochemical conversion of human faeces was carried out by defining a cylinder with dimensions to allow for the necessary residence time of the pellets inside the combustor, according to empirical observations made during the preliminary tests. Caps were manufactured for ports that were not used in each case. The combustion chamber designed to be made of Macor™ can be seen in Fig. 4. This image also includes a picture of the actual piece so the size of this item can be better visualised. The dimensions of the glass-ceramic cylinder, which acts as the combustion chamber, are: outlet diameter of 50 mm, inlet diameter of 37 mm, and height of 205 mm. Additionally, a few holes were machined into the piece:

– Three long ports (length:37 mm and height:3 mm) to insert the grates which hold the fuel pellets, and will establish the physical barrier between zones;
– Five circular ports of 2 mm diameter, to insert thermocouples;
– Five circular ports of 5 mm diameter, three of which are used to feed gaseous streams; and two for instrumentation (i.e. spark igniter and ash disturber);
– Three circular ports of 6 mm diameter to supply gaseous streams;
– Upper lid, which has a 20 mm circular port for fuel feeding, and a 6.4 mm circular port for exhaust outlet;
– Bottom lid, which has a 20 mm circular port for ash disposal, and two 6.4 mm circular ports for exhaust outlet and recycled gas inlet.

**Fig. 5:**
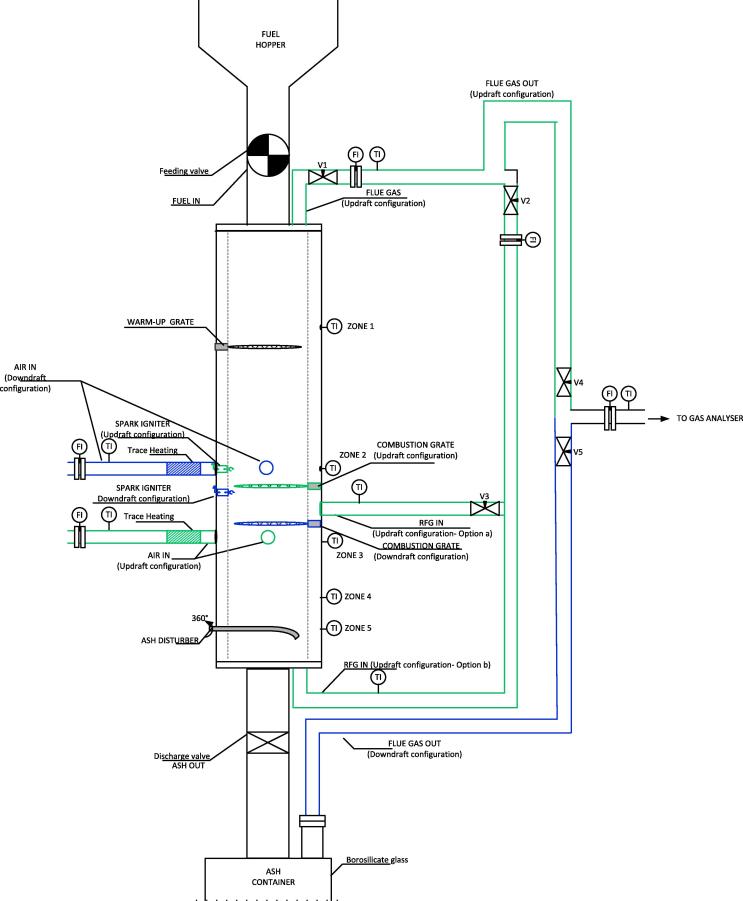
Conceptual design of the prototype able to operate in multiple operation modes (green: updraft; blue: downdraft). Nomenclature used for thermocouples included. (For interpretation of the references to colour in this figure legend, the reader is referred to the web version of this article.)

The diagram shown in Fig. 5 presents how the different modes of operation can be achieved using the same device, by exchanging the inlet and outlet ports for the gaseous streams and instrumentation (i.e. spark igniter, combustion grate, etc.).

The fuel hopper (diameter: 70 mm, height: 75 mm, angle: 60°) was designed to have the capacity to contain the fuel needed for two hours of operation; similarly to the ash container. However, the ash container (inlet diameter: 60 mm and height: 110 mm) was designed considering an additional volume to allow for the gas to pass through it for the tests performed in the downdraft operation mode.

**Fig. 6:**
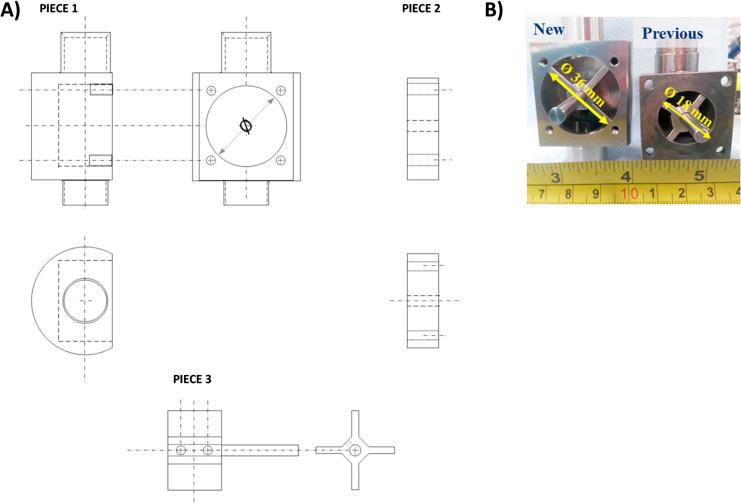
Rotary feeding valves in-house designed and manufactured. (A) Schematics used for design of the three pieces of the valve; (B) actual valves manufactured.

The rotary valve associated with the feeding system, used in the first instance, presented some jamming problems when using human faeces pellets. Thus, a second rotary valve was designed for which the cylindrical internal body was doubled, as can be observed in Fig. 6, to enhance the flow of the pellets.

The tolerances applied to those elements where different materials were used (e.g. fuel feeding stainless steel pipeline connected to the body of the combustion chamber in Macor™). The changes in length of the elements, which are related to the tolerances that must exist in the unions where the joining of different materials occurs, were calculated by means of Eq. (1).

(1) $$\Delta \text{L}={\text{L}}_{0}\cdot \alpha \cdot ({\text{T}}_{1}-{\text{T}}_{0})$$

where ΔL is the change in object length (m), L_0_ is initial length of object (m), α is the linear expansion coefficient (m/m·°C), T_0_ is the initial temperature (°C), and T_1_ is the final temperature (°C). For each joint, two tolerances were calculated: one corresponding to an average increase of temperature (ΔT_aver_), and the other a value for the maximum tolerance needed by applying the maximum increase in temperatures observed during the preliminary tests (ΔT_max_). Some examples of the values calculated are presented in Table 5.

**Table 5:** Data considered for expansion of materials and examples.

Data considered for expansion of the materials’ calculation						
Thermal expansion coefficient, α (m/(m °C)			Location of joint			
			Top and bottom lids of ceramic body		Ceramic body (combustion chamber)	
SS 316	Macor™	BG	ΔT_aver_ (°C)	ΔT_max_ (°C)	ΔT_aver_ (°C)	ΔT_max_ (°C)
16·10^-6^	9.3·10^-6^	4·10^-6^	400	550	500	750

Examples of maximum expansions calculated	

Joint	ΔL_max_ (mm)

Ceramic body	1.2
Shaft of turning grates	0.024
Top lid of ceramic body connected to pipe from rotary valve	0.13

SS: Stainless steel; BG: Borosilicate glass.

**Fig. 7:**
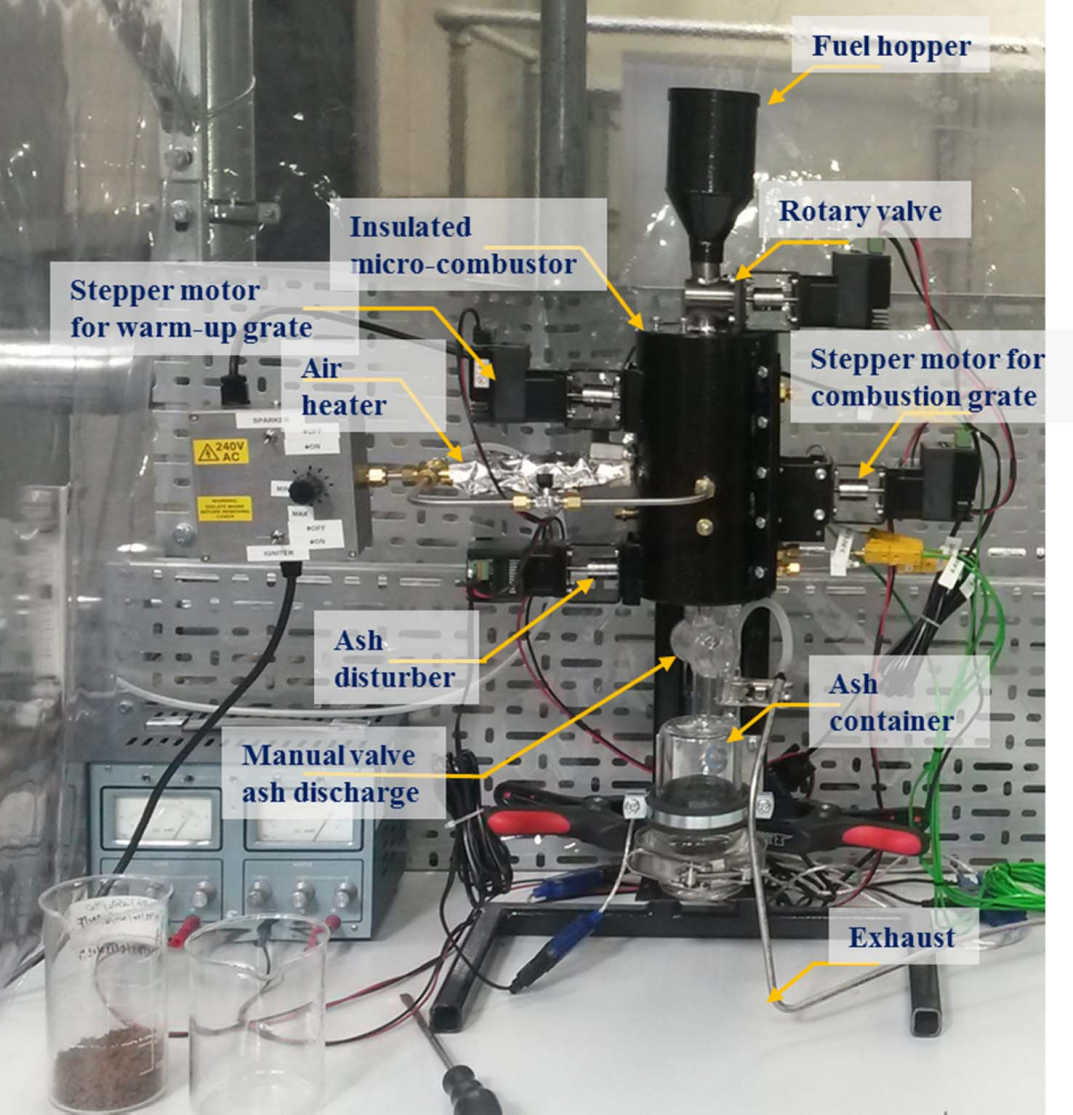
Image of the experimental set-up of the micro-combustor prototype using downdraft mode.

Various parameters are measured and recorded through a data acquisition system (e.g. ignition temperature, temperatures at different sections of the combustion chamber, and composition of the exhaust gas generated). The combustion chamber is divided into three zones, each of them separated by a grate; these grates can turn automatically to control the time that a pellet of fuel remains in each zone of the combustor (drying/warm-up, combustion or char-to-ash). The control system that governs these residence times is one of the aspects studied and optimised with the micro-combustor prototype. This system acts on the fuel feeding rate as well. These mobile parts are triggered by four stepper motors (STM17Q-2AN, Applied Motion Products™) which are connected to the computer from which they receive the control signals. These routines were varied to study the control sequence that gives rise to the best operation in terms of sustainability of the combustion. In addition to this, analyses of the ash generated were carried out to characterise their composition as a consequence of the operating conditions tested. Fig. 7 shows the experimental set-up for the downdraft operation of the micro-combustor.

*Experimental procedure*

The prototype was tested with simulant faeces pellets and human faeces pellets.

The upstream or downstream operation mode of the micro-combustor was set up prior to the start of each test. For this, the inlet/outlet ports are connected accordingly and the valves arranged to permit the pathways of the inlet air and the exhaust. The speed of the rotary valve for fuel feeding and the ash disturber were established using the Python-control interface, as well as for changing the position of the warm-up and combustion grates. The ports of the reactor, which were not in use for a specific test, were sealed using threaded ceramic caps.

At the beginning of the test, the heating element that pre-heats the air was switched on to uniformly heat the reactor steadily at 6 °C/min. This is crucial to avoid strain problems at the points where stainless steel and Macor™ are joined. Once the combustion chamber was preheated to the temperature at which ignition was studied in that specific test, a first batch of fuel was fed into the first zone (warm-up) of the micro-combustor by starting the stepper motor connected to the rotary valve and the stepper motor connected to the first grate. The fuel pellets were accumulated in the second zone (combustion) for the first phase of the test. The air stream was continuously pre-heated until the ignition happened (denoted by a sharp increase in the temperature shown by the thermocouple located in ZONE 2, see Fig. 5). After ignition, the heating device was stopped to assess the self-sustainability of the combustion reaction. The stepper motor linked to the combustion grate was started after a specified time of operation which can vary from 2 min to 40 min depending on the experiment, to ensure that there is a sufficient bed of fuel and ash to act as a heat buffer. The ash disturber was not activated until steady operation had been achieved, which is defined by a tendency of the temperature profiles to stay horizontal.

The temperatures at the different zones of the combustion chamber were recorded, as well as the air flowrates in and out of the micro-combustor. The exhaust gas was passed through three bubblers for condensation of water vapour and tars, and conveyed to the gas analyser. The gas composition for CO_2_, CO, O_2_ and H_2_ was measured and recorded at every second interval via a data logging system. During the test, the ash generated is visually monitored to check that has an acceptable degree of burnout. The presence of char was indicative that the residence time in the char-to-ash zone needed to be increased. At the end of the experiment, the remaining ash was collected, weighed and stored for later analysis (i.e. residual carbon and ash composition).

*Experimental plan*

An experimental plan was projected to study the different aspects of the operation of the micro-combustor for optimal operating procedure that would give rise to a steady and self-sustained thermal conversion of human faeces. The plan outlined had the following stages, and was duplicated for using simulant and real faeces:

1. Assessment of optimal fuel pellet size.
2. Assessment of mass of initial fuel batch fed to promote continuation of combustion after ignition.
3. Assessment of grate sequence. This control sequence affects the residence time of the fuel in each zone of the reactor.
4. Selection of optimum air to fuel ratio.
5. Selection of optimum ignition procedure (using the designated air-to-fuel ratio).
6. Identification of the main operation parameters to allow for a self-sustained operation.

## 3 Experimental results and discussion

### 3.1 First testing campaign with the micro-combustor prototype

Experiments using simulant and real faeces were carried out using the updraft mode without recycling of the flue gas. For this set of experiments, the temperature across the reactor was measured but exhaust gas composition was not recorded. These tests were informative as they showed the optimal mass of fuel to feed for the initial batch, rotation sequence for grates 1 and 2, optimum air to fuel ratio, and part of the ignition procedure. The main outcomes concluded from these tests were:

– Ignition temperature for sustained fuel ignition process and steady-state operation of the reactor was set between 220 °C and 240 °C.
– Minimum initial fuel feed of 7 g was required to act as a heat buffer for continuous ignition of newly added fuel.
– Fuel residence time and subsequent conversion process time depended on the initial combustion temperature at which the fuel was introduced.
– Optimum air flow for fuel ignition was between 7 and 8 L/min and this corroborates the theoretical estimates.
– Further analysis is needed at full optimum condition testing to investigate if a lower minimum combustion temperature can be achieved.

**Fig. 8:**
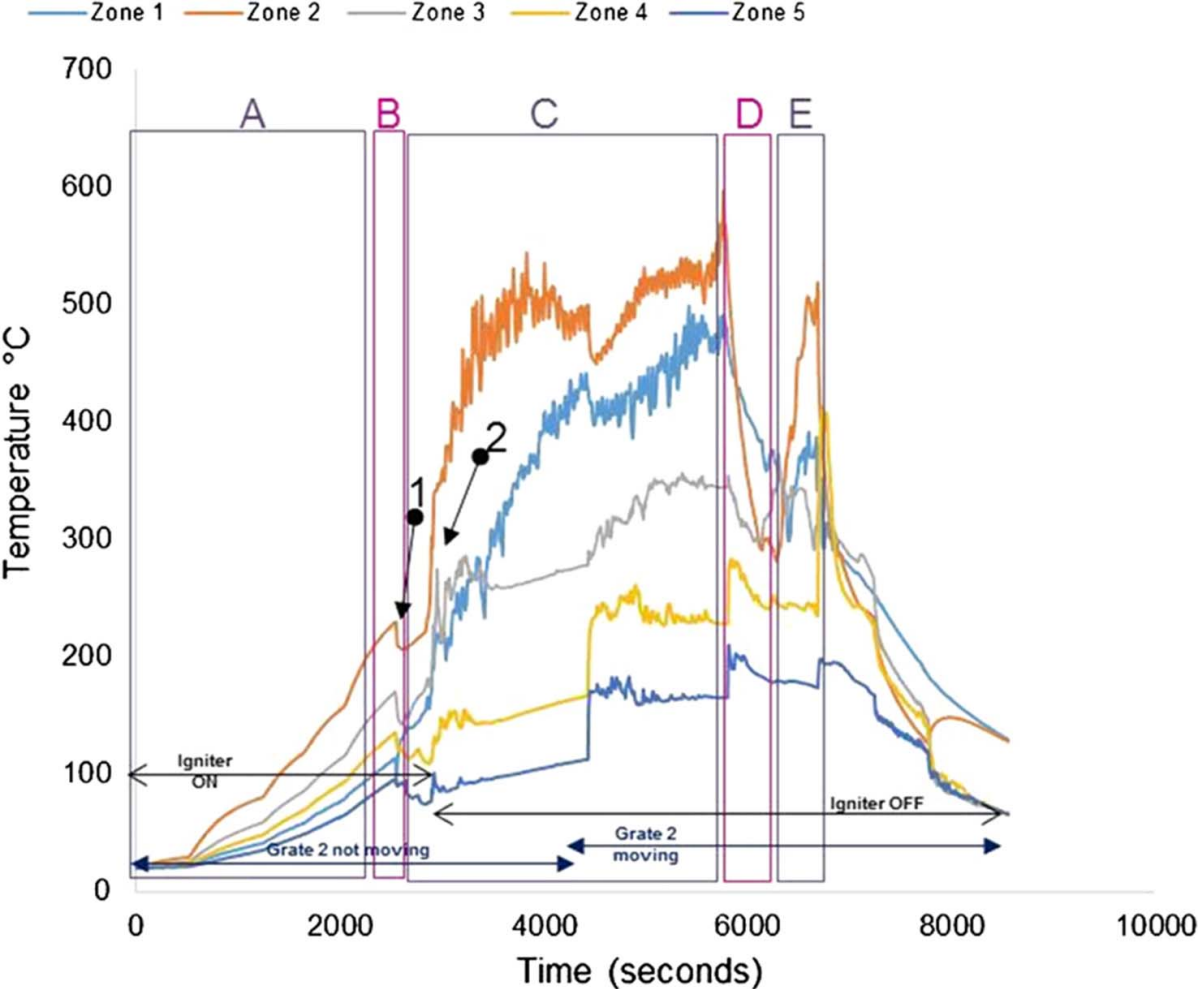
Real faeces; updraft mode; 1.2 g/min; 8 L/min air; test starts at t: 2530 s; test duration: 4200 s; grate 1 turned from t ∼ 2800 s once every 40 s; grate 2 turned at t ∼ 6900 s.

**Fig. 9:**
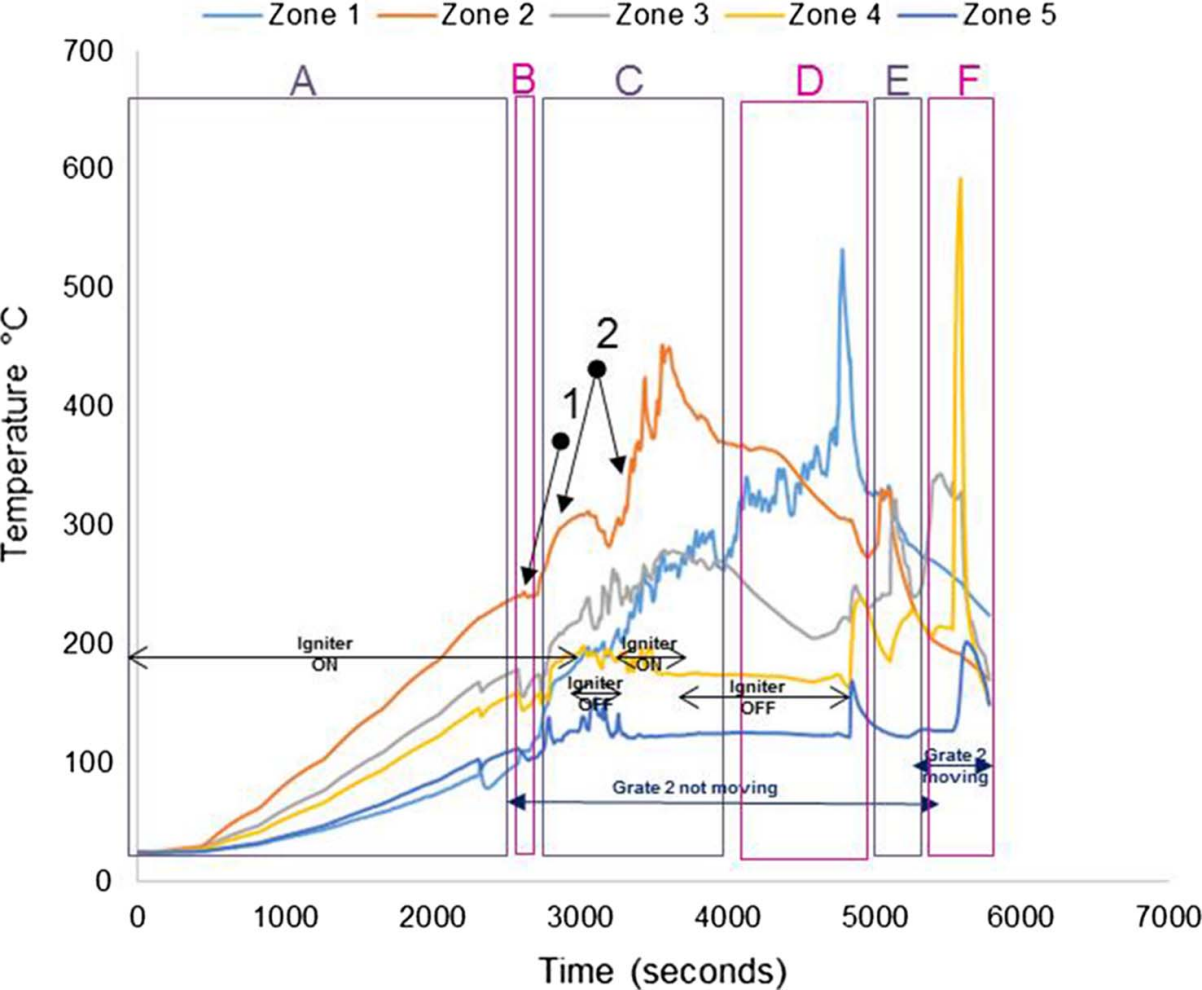
Simulant faeces; updraft mode; 1.38 g/min; 8 L/min air; test starts at t: 2750 s; test duration: 2940 s; grate 1 turned from t ∼ 2800 s once every 40 s; grate 2 turned at t ∼ 5800 s. (Referred to as Test 1 in Table 7).

Fig. 8, Fig. 9 show the temperature profiles inside the reactor for some of the tests conducted during the first testing campaign. Details of the experimental conditions are provided in the caption of each figure. The test duration was logged from the instant at which the first batch of fuel was fed into the reactor. The pre-heating period of the unit is included and denoted as (A); where it can be seen how the temperature profiles increase steadily at the different locations inside the micro-combustor. The process has been classified into the following stages:

(A) Pre-heating of the unit using air igniter (without fuel inside);
(B) First batch of fuel fed (7 g) with air igniter on;
(C) Ignition of the fuel. The fuel was fed at a continuous rate of ∼1.2 g/min with the air igniter still on until ignition of the fuel was achieved. The ignition is characterised by a sharp increase in temperature in Zone 2 (orange line). The air igniter was switched off once the pre-set temperature to be studied (in the range of 300–400 °C) had been reached; during this stage, the combustion or gasification, depending on the test, of the fuel was ongoing in Zone 2;
(D) Migration of the combustion or gasification process to a different zone from Zone 2 (denominated earlier as the combustion zone);
(E) Migration of the combustion or gasification process to another zone from that specified in stage D);
(F) Migration of the combustion/gasification process to another zone from that specified in stage E).

Ideally, the migrations of the combustion or gasification of the fuel pellets should not occur; the burning process should remain in Zone 2. However, at this stage of the development of the prototype, the control routines for turning the grates to achieve a self-sustained operation were still under study. Similar to the observations in this study, Plis et al. [24] showed that the temperature profile in an updraft gasifier with 20 kg/h biomass capacity varied and progressively increased across the different zones. The upper part of the gasifier had the highest temperatures with values varying between 800 and 1000 °C during steady operation. This is due to the counter flow of the product gas across the bed with the hottest part of the flame at the upper region of the reactor. Fig. 8, Fig. 9 show a slightly lower temperature profile to this range. This could be due to variation in reactor geometry, biomass capacity and type, simulant and real faeces in this case.

In each of the stages described above, there were milestones commonly observed for all the tests

(1) Drop of temperature due to first fuel feeding.
(2) Ignition of the fuel.

Other operating parameters or conditions occurred during the tests are included in the graphs for a better understanding of the process, as most of them had an effect on the subsequent temperatures and/or gas composition measured:

– Instant at which the air igniter was on/off.
– Instant at which grate 2 started turning at a certain frequency.
– Instant at which the ash disturber was activated.
– Instant at which the fuel feeding valve was jammed so it was not delivering any fuel.

The test presented in Fig. 8 was the trial with maximum duration achieved during the first testing campaign with the micro-combustor prototype.

Fig. 9 shows the temperature profiles for one of the first trials carried out with the prototype in updraft configuration mode. The adequate temperature to switch off the igniter and the possibility of fuel re-ignition were studied in this trial and, consequently, periods with the igniter switched on and off can be observed.

#### 3.1.1 Modifications resulting from first results

The main issues, encountered during the tests that were run during the first campaign, are listed in Table 6, together with the solutions implemented for them:

**Table 6:** Operational issues observed during first campaign's tests and solutions applied.

Issue observed	Practical solution
Rotary feeding valve was not big enough for the pellets to pass by without having jamming problems	Design and manufacture of a bigger valve; internal diameter of the body of the valve increased from 18 mm to 36 mm. This enhanced the fuel feeding although the problem has not been fully solved when looking at prolonged tests (over 70 min)
Funnel connecting the fuel hopper with the rotary valve was causing bridging of the fuel pellets	Cylindrical bottom section of the fuel feeder was shortened by 15 mm
Ash blocking bottom part of the ceramic reactor after 30 min of operation	Installation of additional axial ash disturber
Tars condensation in exhaust line	Installation of two bubblers in an iced bath and one with rock wool in the gas sampling line. Also wider connector (previous: 5 mm, new: 12 mm) between ash container and exhaust line
Exhaust not flowing through gas sampling lines, passing instead by the rotary feeding valve; this generated a char cake in the body of the valve, blocking the system	Installation of a Coanda air mover at the end of the exhaust line to induce the flow in this direction

### 3.2 Second testing campaign with the micro-combustor prototype

**Fig. 10:**
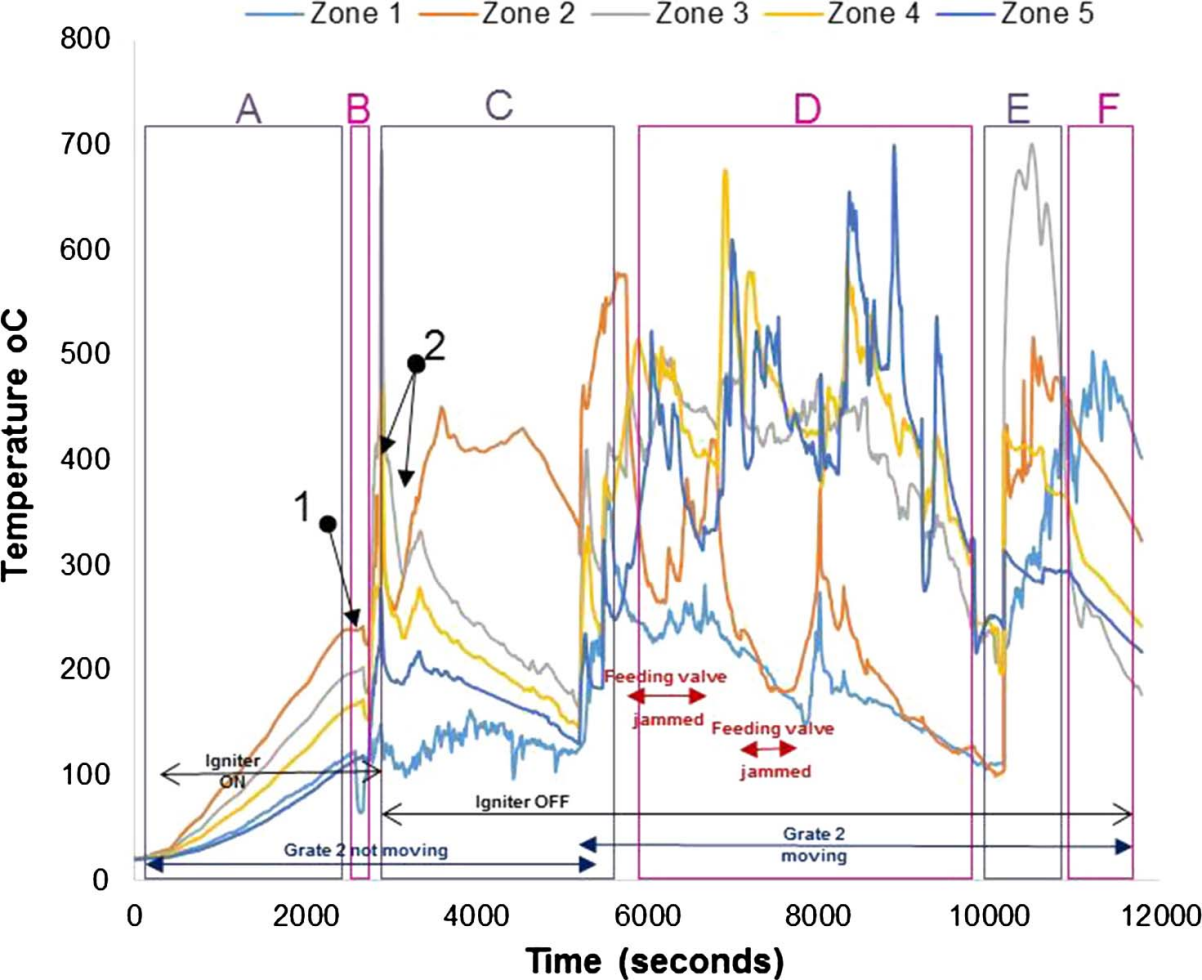
Simulant faeces; downdraft mode; 1.34 g/min; 7.5–8 L/min air; test starts at t: 2600 s; test duration: 7800 s; grate 1 turned from t ∼ 2650 s once every 40 s; grate 2 turned at t ∼ 5300 s. Maximum and average temperatures in gasification/combustion zone: 701 °C and 300 °C.

**Fig. 11:**
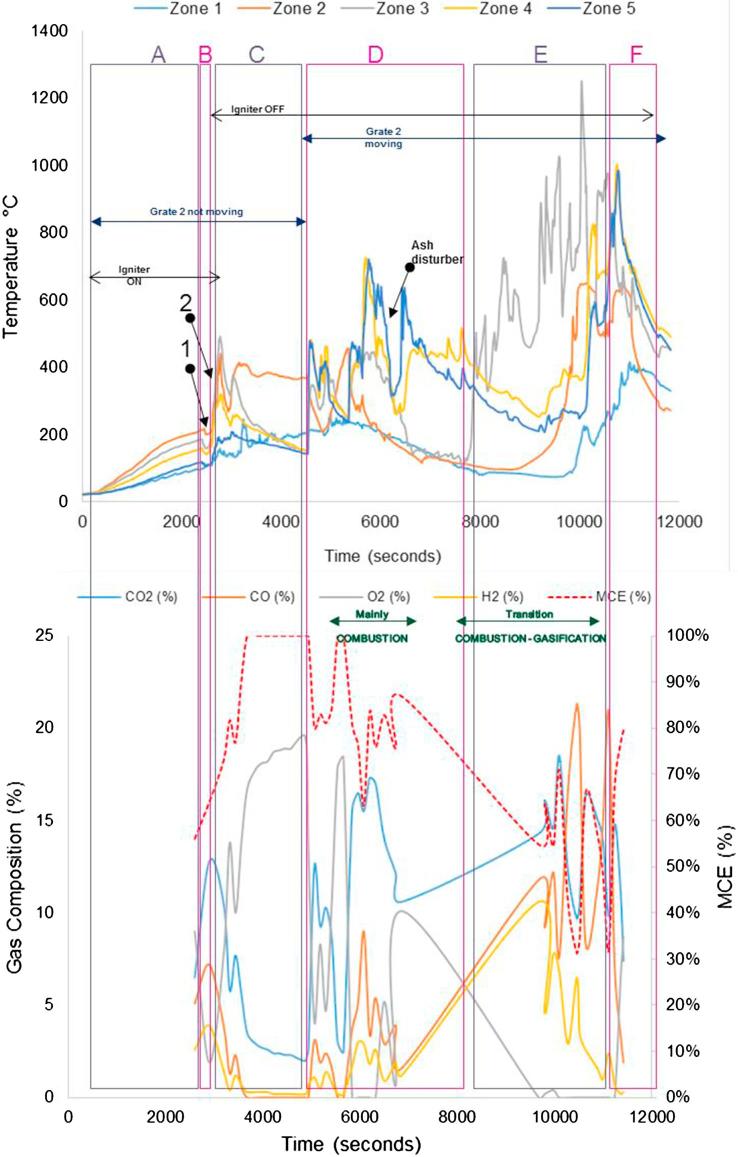
Real faeces; downdraft mode; 1.22 g/min; 7.5–8 L/min air; test starts at t: 2595 s; test duration: 9000 s; grate 1 turned from t ∼ 2640 s once every 40 s; grate 2 turned at t ∼ 5300 s. Maximum and average temperatures in gasification/combustion zone: 1226 °C and 441 °C. MCE: modified combustion efficiency. (Referred to as Test 4 in Table 7.)

**Fig. 12:**
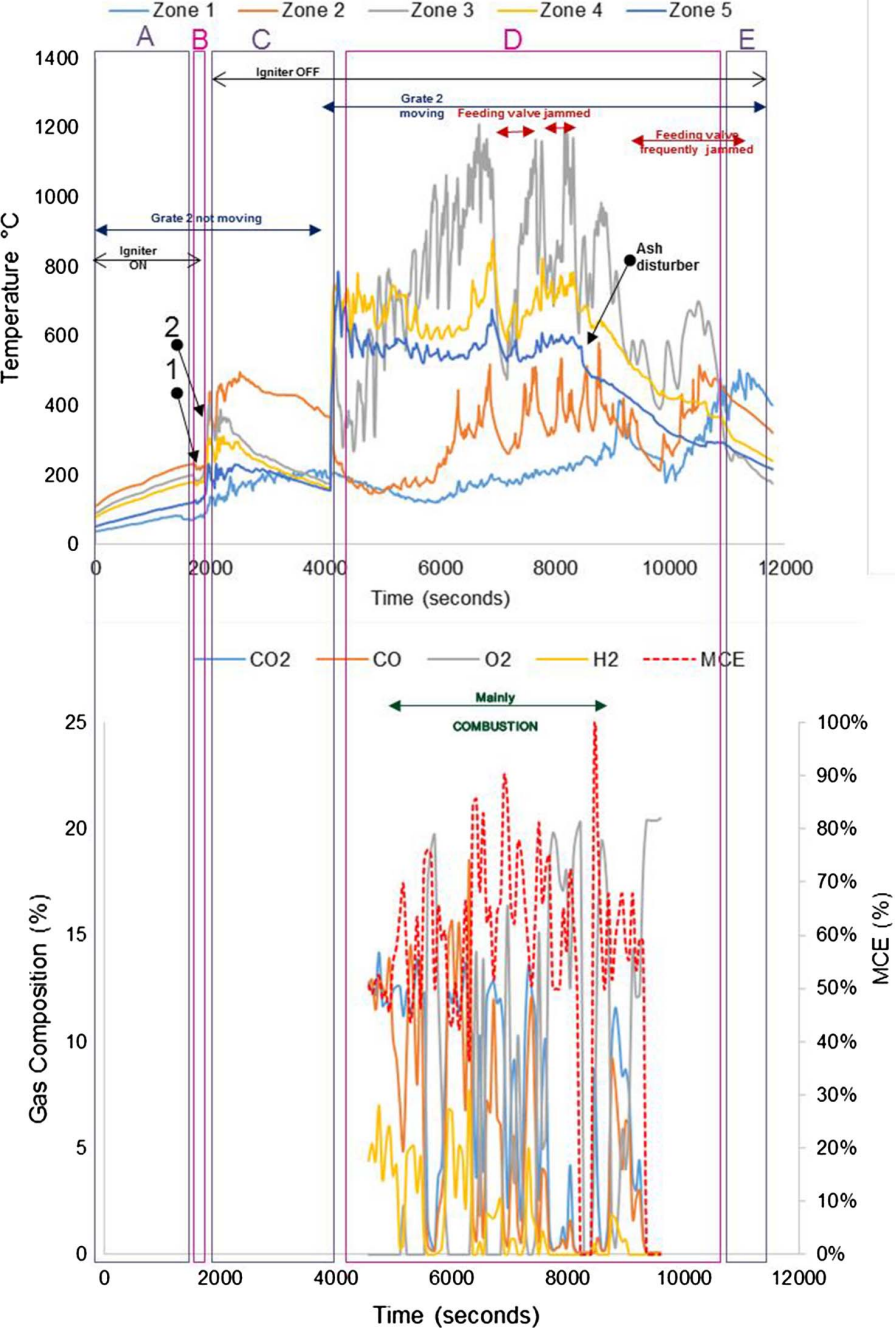
Real faeces; downdraft mode; 1.20 g/min; 7.5–8 L/min air; test starts at t: 1920 s; test duration: 10080 s; grate 1 turned from t ∼ 1980 s once every 40 s; grate 2 turned at t ∼ 4100 s. Maximum and average temperatures in gasification/combustion zone: 1211 °C and 574 °C. MCE: modified combustion efficiency. (Referred to as Test 2 in Table 7.)

**Fig. 13:**
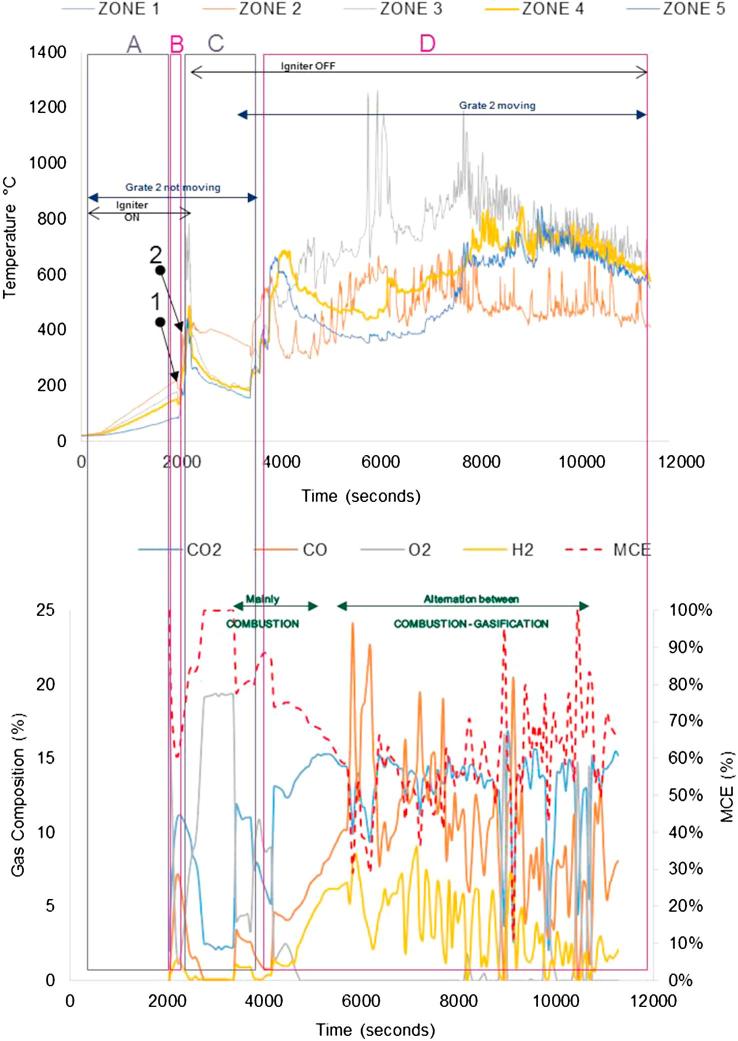
Real faeces; downdraft mode; 1.9 g/min; 7.5–8 L/min air; test starts at t: 1950 s; test duration: 9540 s; grate 1 turned from t ∼ 2000 s once every 40 s; grate 2 turned at t ∼ 3900 s. Maximum and average temperatures in gasification/combustion zone: 1240 °C and 668 °C. MCE: modified combustion efficiency. (Referred to as Test 5 in Table 7.)

The tests performed during the second campaign helped to give responses to those objectives set up as part of the experimental plan that could not be answered during the first campaign (see objectives 1, 5, 6), such as pellet size, ignition procedure and main parameters affecting self-sustained operation. Fig. 10, Fig. 11, Fig. 12, Fig. 13 give details of the most illustrative tests performed after implementing the solutions explained in Table 6. The nomenclature used to classify the stages during a test, and to highlight changes in operating conditions or one-off facts is the same as described in Section 3.1.

The impact on the process temperature profiles when the normal feeding of fuel was interrupted, due to valve blockage, can be observed in Fig. 10. This happened around t ∼ 6000 s, and caused the combustion front to move down within the reactor (from Zone 2 to Zones 4 and 5).

For the experiments plotted in Fig. 11, Fig. 12, Fig. 13, the modified combustion efficiency was calculated by means of Eq. (2) as an indicator of the degree of combustion occurring in the reactor.

(2) $$\mathrm{MCE}\;(\%)=([{\text{CO}}_{2}]/([{\text{CO}}_{2}]+[\text{CO}])\cdot 100$$

Note that some problems were experienced with the logging system for gas composition during the first half of the test shown in Fig. 12. These temperature profiles show the issues experienced when the fuel was blocking the feeding valve which had a direct effect on the temperatures, i.e. they decreased abruptly – an effect that sometimes could not be overcome as it happened towards the end of the test. This test presented mainly combustion, although some gasification was occurring, as it can be seen in those periods where higher levels of H and CO were produced (e.g. 6000–6600 s).

For the gas composition recorded during the test presented in Fig. 13, it must be considered that the high values of oxygen logged in the period between 2500 s and 3500 s are because the gas sampled was air; some changes had to be carried out in the gas sampling line for the first moments of operation, during which the analyser was measuring ambient air.

From the graphs presented in Fig. 10, Fig. 11, Fig. 12, Fig. 13 there are some remarks that are common for all of them:

– Initial drop in temperature is quite subtle, when the first batch of fuel is fed into the reactor.
– When grate 2 is turned for first time, there is a decrease in the temperature logged at location Zone 2 (see Fig. 5) and an increase in the temperature located in the ash zone (Zones 4 and 5).
– When the fuel inlet valve was becoming blocked for periods longer than 2–3 min, a drop in the temperature profiles across the reactor could be noticed.
– When the ash disturber is actioned, a decrease in the temperature logged for the location Zone 5 could be observed.

The residues, mainly ash, were collected and weighed after the tests. Occasionally, some char was observed in the residues due to incomplete burnout of the last batch of sample fed into the reactor.

The analyses carried out to measure the unburnt carbon remaining in the ash showed results in the range between 14 wt.% and 24 wt.% in the ash collected from the combustion of real faeces. These analyses were performed using the loss-on-ignition (LOI) approach [25] to determine the residual carbon content in the leftover ash.

The analyses undertaken using the environmental scanning electron microscope (ESEM) provided information about the elemental composition of the ash generated. These data are presented in Table 7. The cases described as ‘Global’, in this table, were those analyses undertaken considering a representative area of the sample; the analyses described as ‘Detail’ belong to particles that presented morphological differences from the rest of the sample.

**Table 7:** Elemental composition (wt.%) of ash collected from various tests; details given of magnifications applied in the ESEM for each case (×250: 250 times magnified; ×500: 500 times magnified; ×1000: 1000 times magnified; ×2000: 2000 times magnified); SF: simulant faeces; RF: real faeces.

Spectrum	Test	Description	C	O	F	Na	Mg	Al	Si	P	S	Cl	K	Ca	Ti	Mn	Fe	Zn
1	SF Test 1	Global (×250)	3.97	36.94	0.22	4.89	0.66	–	0.32	15.02	0.09	4.72	3.07	30.09	–	–	–	–
2	SF Test 1	Detail 1 (×500)	5.14	35.15	0.27	4.98	0.61	–	0.31	15.14	0.09	4.9	2.85	30.55	–	–	–	–
3	SF Test 1	Detail 2 (×1000)	10.76	35.53	0.58	3.62	0.56	–	0.20	13.25	0.18	3.8	3.08	28.43	–	–	–	–
4	RF Test 2	Global (×250)	0.79	40.92	0.83	0.94	11.9	–	0.58	11.65	0.75	0.24	12.72	18.11	–	–	0.57	–
5	RF Test 2	Detail 1 (×500)	8.77	33.76	0.20	1.06	4.31	–	0.58	12.07	0.76	0.76	15.72	21.32	–	–	0.70	–
6	RF Test 2	Detail 2 (×1000)	15.68	34.04	0.32	0.77	4.41	0.32	0.6	12.01	0.51	0.50	10.46	19.63	0.26	–	0.51	–
7	SF Test 3	Detail 1 (×1000)	51.67	22.75	0.30	1.75	0.22	–	0.10	5.65	0.21	3.29	2.08	11.98	–	–	–	–
8	RF Test 4	Global (×250)	4.83	39.98	0.38	0.51	6.31	0.18	0.69	13.01	0.28		8.73	23.92	–	–	0.47	0.70
9	RF Test 4	Detail 1 (×500)	19.45	32.73	0.50	1.02	3.66	–	0.51	10.65	0.76	0.56	11.4	17.51	–	–	0.43	0.84
10	RF Test 4	Detail 2 (×1000)	11.24	33.29	0.20	1.14	3.98	–	0.38	11.5	1.61	0.44	13.72	21.78	–	0.26	0.47	–
11	RF Test 4	Detail 3 (×1000)	12.08	33.31	0.50	1.34	4.57	0.91	0.89	11.31	0.47	0.53	15.86	17.10	0.15	0.18	0.43	0.38
12	RF Test 5	Global (×250)	9.83	33.86	0.47	0.78	4.34	0.15	0.64	12.70	0.72	1.37	14.09	19.46	–	–	0.34	1.24
13	RF Test 5	Detail 1 (×500)	23.31	29.24	0.34	0.57	3.67	0.17	0.40	9.22	1.23	1.68	13.42	15.65	–	–	0.45	0.64
14	RF Test 5	Detail 2 (×1000)	10.54	36.44	0.63	0.67	5.00	0.33	0.45	13.95	0.38	0.49	6.83	23.92	0.15	–	0.22	–
15	RF Test 5	Detail 3 (×2000)	12.63	30.18	0.43	0.70	3.16	0.06	0.29	8.16	4.93	1.16	9.7	27.74	0.17	–	–	0.68

The carbon levels obtained are in the range between 4 wt.% and 12 wt.%; except for test 3 where no optimal combustion could be achieved during operation of the bench-scale facility which resulted in a higher presence of unburnt carbon (51.67 wt.%). Calcium, potassium, and phosphorus can be observed to be the main elements found in all the cases after oxygen. There are specific cases, looking at ashes generated from burning real faeces, which show in their composition iron, zinc, aluminium and titanium.

**Fig. 14:**
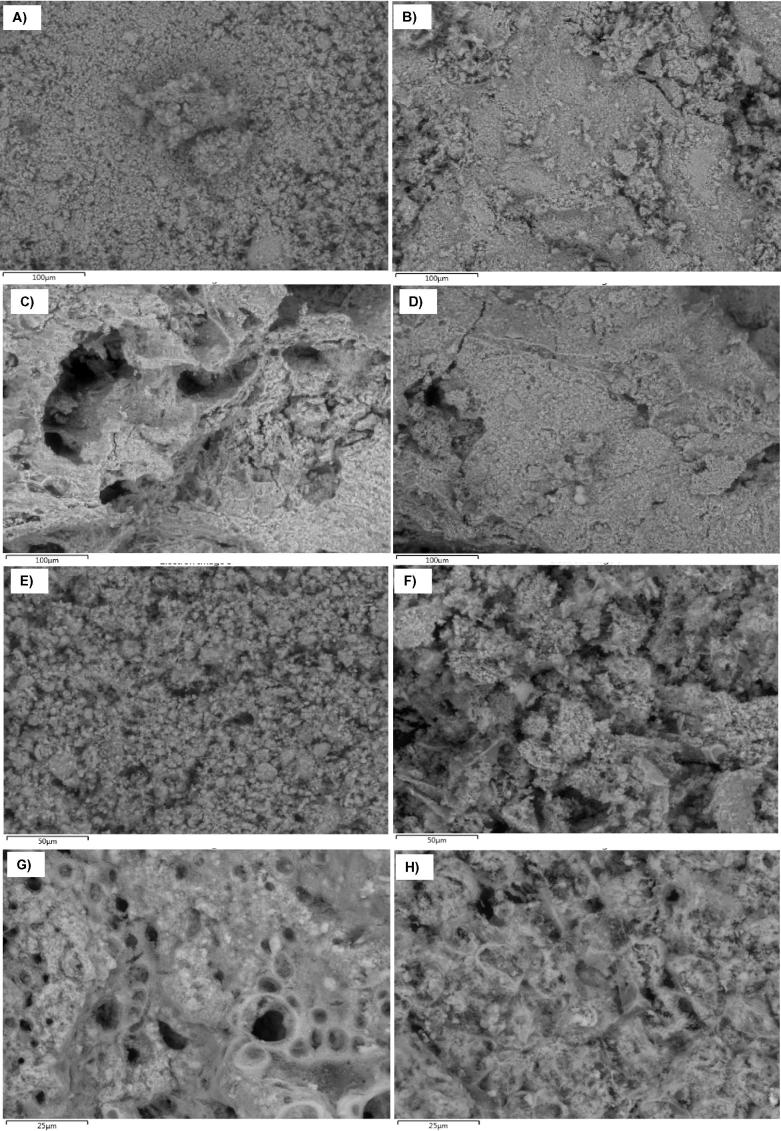
ESEM images of ashes generated at the bench-scale gasifier-combustor. (A) SF Test 1- ×250 times magnified; (B) RF Test 2- ×250 times magnified; (C) RF Test 3- ×250 times magnified; (D) RF Test 4- ×250 times magnified; (E) SF Test 1- ×500 times magnified; (F) RF Test 2- ×500 times magnified; (G) RF Test 4- ×1000 times magnified; (G) RF Test 5- ×1000 times magnified. SF: simulant faeces; RF: real faeces.

Some of the images captured, using the ESEM technique, are presented in Fig. 14. The difference in ashes generated by simulant and real faeces can be appreciated comparing Fig. 14(A) with (B–D), where the ash from RF shows a more fibrous and complex structure. For each sample at least four areas were evaluated from which only the most interesting cases have been included in Fig. 14. Some of the images show the details of specific particles observed during the analyses, which presented different morphologies and so were analysed individually and captured using higher magnifications (see Fig. 14(E)–(H)). Note the fine structures detected in Fig. 14(H) which are at submicron levels; this must be considered in the safety procedure when working with this type of ash, where masks with appropriate filters should be used.

#### 3.2.1 Optimal operating procedure

The optimal operation procedure was defined after performing various types of test, each of them aimed at studying a different parameter and its effect on the process. The types of test carried out have already been listed in the subsection dedicated to the description of the test campaign. The conditions that favour the continuous gasification/combustion of human faeces in the designed micro-combustor are specified as follows:

1. The size that permits the appropriate feeding through the rotary valve is <5 mm for simulant faeces and <7 mm for real faeces.
2. The mass for the initial batch fed to the reactor must be 7 g.
3. The frequency of rotation for the first grate (warm-up zone) is once every 40 s and once every 180 s for the second grate (gasification/combustion zone).

Reed et al. [26] identified the flexible advantages of open-core design of fixed-bed downdraft gasifier over conventional imbert or throated designs. Some of the disadvantages of the conventional designs include: fuel pre-treatment requirement due to hearth constrictions in imbert gasifiers, complex heterogeneous reactions between the fuel and air that makes it difficult to understand, model and design the processes associated. Stratified “open-top or topless” gasifier on the other hand are considered more flexible due to uniform passage of air and fuel to the flaming pyrolysis, char reduction and inert char zone. The absence of top biomass fuel enables the control of fuel into the system, the fuel burn rate in the flaming pyrolysis and temperatures in the oxidation/reduction zones while the inert char zone acts as a heat buffer and further insulation. These interactions and processes make it relatively easy to model, understand and develop. For instance, Di Blasi et al. [27] recently developed a mathematically-model of a stratified wood downdraft. The study highlighted the potential of achieving multiple stabilisation of the flame-front for gasification of wood biomass. The findings in this study thus confirm that the open-core configuration can be achieved at a micro-scale and the multiple stratified zones aided by double grates can enable the pre-heating, drying and decomposition of the solid fuel. More so, the controlled ash zone and retaining of the ash can aid any heterogeneous char conversion processes.

4. The optimum air to fuel ratio is defined as 6.4 L_air_/g_fuel._ This value corresponds to an excess of air of 6% over the stoichiometric calculated without considering the oxygen contained in the fuel.

Several studies [18], [28], [29], [30] have examined the effect of air-to-fuel ratio on gasification/combustion efficiency and for various reactors and fuels. In all these studies, it is generally agreed that there is an optimum air-to-fuel ratio for fuel conversion, although a reactor can be designed to operate a variety of fuels. For instance, Gai et al. [30] showed that bed temperature distribution is subject to equivalence ratio, consequently air-to-fuel ratio. Here, increasing equivalence ratio, which is observed with increased excess air or oxygen concentration, favours heat release and further conversion of CO to CO_2_. Other studies such as Sharna et al. [31] and Erlich et al. [32] also showed that pellet size or geometry and operating conditions such as blower characteristics, gas flow conditions, char bed porosity can affect reactor dynamics and influence the overall performance of the gasifier. As such, thorough sensitivity analysis of operating conditions of the reactor is a paramount experimental investigation, particularly for newly designed reactor. This is currently out of scope of this work.

5. For the ignition, the first batch must be fed when the temperature above the second grate, Zone 2 (see Fig. 5) reaches 200–220 °C. The heating system must be kept on until the temperature in Zone 2 reaches above 400 °C, at which time the igniter is switched off for the rest of the test. The spark device included as part of the ignition system proved not to be necessary as long as the combustion chamber had been pre-heated.

Monhol and Martins [33] also reported the ignition temperature of dried human faeces at about 220 °C. Ignition was achieved by exposing the sample to radiative heat flux density of 25–30 kW/m^2^.

6. Several aspects have been acknowledged to contribute to the stability of the thermal conversion of the fuel pellets:
  6.1. It is crucial that the fuel feeding is not delayed for longer than 2 min, as otherwise the temperature inside the reactor drops significantly, which can lead to preventing the thermochemical conversion from taking place. This is a consequence of the scale of the process, as the fuel rate (1.2 g/min) is the minimum that has been tested to promote continuous operation.
  6.2. The speed of the rotary valve feeding the fuel must be high enough not to allow the fuel to start to react/oxidise when it is at the valve body. This can lead to char formation; this char might then be stuck to the valve walls, creating a cake which causes the obstruction of the valve. This situation could be solved by using a double valve system, in which the first valve provides the required fuel flowrate (at low speed) and the second valve, which is connected to the reactor (so it ‘sees’ higher temperatures), can rotate at a higher speed – avoiding the char formation inside. Judex et al. [34] alluded to the challenges of controlling the feed of sewage sludge in a pilot-scale gasification plant due to counter flow of syngas across the fuel feed system, such that progressive deposition of organic and inorganic impurities in the syngas leads to blockage of the fuel in the hopper. Thus, careful design of the fuel feed system was suggested with appropriate process control and gas purification to ensure maximum plant efficiency. To minimise fuel contamination, Reed et al. [26] described a partitioning of the fuel feed system using lock hoppers and valves. These systems need to be further designed and developed as current design do not ensure accurate sealing of the gas path from the fuel feed system. For instance, channelling of the rotary valve can be minimised by using a double valve.
  6.3. The second grate (gasification/combustion zone) must be kept stable for the initial 35–40 min of the process to ensure that there is a sufficient heat buffer. After this initial delay in the second grate, subsequent times involve the turning of the second grate every 180 s – a process where steady-state operation is reached.
  6.4. The ash disturber must not be activated until some accumulation of ash is observed at the char-to-ash zone (usually after 50–60 min of operation).
  6.5. Once steady-state operation is reached inside the reactor, the Coanda air mover must be bypassed because the gasification/combustion process generates enough pressure inside the reactor to make the flue gas circulate through the sampling gas line.

## 4 Conclusions and further recommendations

A micro-combustor prototype has been designed and commissioned successfully for the thermochemical conversion of human faeces as part of the development of next generation sanitary systems that can work without connection to external water, energy or sewerage systems.

The most challenging aspect of the design of this testing unit was to achieve a configuration that promoted a self-sustained combustion, considering the scale of the process, between 0.43 and 1.2 g/min of dry human faeces. These flowrates were defined simulating two operation periods of the combustion unit: 24 h/day and 8 h/day. For this, the minimisation of the heat loss was a fundamental aspect to consider. This was done by selecting a ceramic material for the combustion chamber and by sizing the insulation system appropriately. The design of the prototype was carried out in two stages: firstly, performing a number of preliminary tests to gather information about the essential aspects of a combustion process on a lab-scale (e.g. ignition temperatures, residence times required depending on fuel used, maximum temperatures reached, ash characteristics); and secondly, the actual design of the unit considering the lessons learnt during the first phase. The final product of this work is a micro-combustor that can operate in two modes, downdraft and updraft, providing a flexibility of operation which allows for a comprehensive study of the thermal conversion of human faeces at this stage of process and technology development. The intensive test plan carried out with the different versions of the prototype has generated very valuable experimental data to understand and optimise the thermochemical conversion of human faeces through gasification/combustion. It has also set the first milestone at the minimal scale of the process to be self-sustained and promote a continuous operation.

The downdraft mode has been shown to promote high levels of conversion of the fuel, so the heat released from the combustion of the faeces is used to keep the combustion chamber at a high temperature; however, more work needs to be done to optimise the system so more stable temperature profiles are generated along the reactor. Another important aspect to look at, when using the downdraft configuration, is to keep the exhaust line clean enough for the gas to flow. This is due to the intensive tars formation and its posterior condensation along the flue gas pathway, which can have an impact on the gasification/combustion process as a consequence of the pressure conditions inside the reactor (back pressure).

## References

[1] Nussbaumer T. (2003). Combustion and co-combustion of biomass: fundamentals, technologies, and primary measures for emission reduction. Energy Fuels.

[2] Huang X., Rein G. (2016). Thermochemical conversion of biomass in smouldering combustion across scales: the roles of heterogeneous kinetics, oxygen and transport phenomena. Bioresour Technol.

[3] Milani M., Montorsi L., Stefani M. (2014). An integrated approach to energy recovery from biomass and waste: anaerobic digestion-gasification-water treatment. Waste Manage Res: J Int Solid Wastes Publ Cleansing Assoc ISWA.

[4] Werle S., Dudziak M., Grübel K. (2016). Indirect methods of dried sewage sludge contamination assessments. J Environ Sci Health Part A Toxic/Hazard Subst Environ Eng.

[5] Onabanjo T., Patchigolla K., Wagland S.T., Fidalgo B., Kolios A., McAdam E. (2016). Energy recovery from human faeces via gasification: a thermodynamic equilibrium modelling approach. Energy Convers Manage.

[6] McConville J.R., Kunzle R., Messmer U., Udert K.M., Larsen T.A. (2014). Decision support for redesigning wastewater treatment technologies. Environ Sci Technol.

[7] Bill & Melinda Gates Foundation. Reinvent the toilet – strategy overview; 2016. <http://www.gatesfoundation.org/What-We-Do/Global-Development/Reinvent-the-Toilet-Challenge>.

[8] Bill & Melinda Gates Foundation. Water, Sanitation & Higiene- Strategy Overview; 2016. <http://www.gatesfoundation.org/What-We-Do/Global-Development/Water-Sanitation-and-Hygiene>.

[9] Parker A. (2014). Membrane technology plays key role in waterless hygienic toilet. Membr Technol.

[10] Danso-Boateng E., Holdich R.G., Shama G., Wheatley A.D., Sohail M., Martin S.J. (2013). Kinetics of faecal biomass hydrothermal carbonisation for hydrochar production. Appl Energy.

[11] Hanak D.P., Kolios A.J., Onabanjo T., Wagland S.T., Patchigolla K., Fidalgo B. (2016). Conceptual energy and water recovery system for self-sustained nano membrane toilet. Energy Convers Manage.

[12] Muspratt A.M., Nakato T., Niwagaba C., Dione H., Kang J., Stupin L. (2014). Fuel potential of faecal sludge: calorific value results from Uganda, Ghana and Senegal. J Water Sanitation Hygiene Develop.

[13] Yerman L., Hadden R.M., Carrascal J., Fabris I., Cormier D., Torero J.L. (2015). Smouldering combustion as a treatment technology for faeces: exploring the parameter space. Fuel.

[14] Wall H, Gerhard J, Fabris I, Cormier D, Cheng YL, Torero JL. Investigation of self-sustaining smouldering of faeces: Key parameters and scaling effects. In: Dynamic ecolibrium: Sustainable Engineering Society Conference (SENG 2015); 2015. p. 113–21.

[15] Monhol F.A.F., Martins M.F. (2015). Cocurrent combustion of human feces and polyethylene waste. Waste Biomass Valorization.

[16] Afolabi O.O.D., Sohail M., Thomas C.P.L. (2015). Microwave hydrothermal carbonization of human biowastes. Waste Biomass Valorization.

[17] Ward B.J., Yacob T.W., Montoya L.D. (2014). Evaluation of solid fuel char briquettes from human waste. Environ Sci Technol.

[18] Onabanjo T., Kolios A.J., Patchigolla K., Wagland S.T., Fidalgo B., Jurado N. (2016). An experimental investigation of the combustion performance of human faeces. Fuel.

[19] Pollution Research Group – University of KwaZulu-Natal. Selection of synthetic sludge simulant for the Bill and Melinda Gates Foundation’ s Reinvent the Toilet Fair. India; 2014.

[20] PSEC University of Chicago. Physical and mechanical properties of Kovar n.d.

[21] Shirzadi A. (2008). Solid-state diffusion bonding.

[22] Zhang C., Qiao G., Jin Z. (2002). Active brazing of pure alumina to Kovar alloy based on the partial transient liquid phase (PTLP) technique with Ni-Ti interlayer. J Eur Ceram Soc.

[23] Grossman D.G. (1978). Machining a machinable glass-ceramic. Vacuum.

[24] Plis P, Wilk RK. Theoretical and experimental investigation of biomass gasification process in a fixed bed gasifi er 2011;36:3838–45. http://doi.org/10.1016/j.energy.2010.08.039.

[25] Zhao M., Han Z., Sheng C., Wu H. (2013). Characterization of residual carbon in fly ashes from power plants firing biomass. Energy Fuels.

[26] Reed T.B., Das A. (1988). Handbook of biomass downdraft gasifier engine systems.

[27] Di Blasi C., Branca C. (2013). Modeling a stratified downdraft wood gasifier with primary and secondary air entry. Fuel.

[28] Zainal Z.A., Rifau A., Quadir G.A., Seetharamu K.N. (2002). Experimental investigation of a downdraft biomass gasifier. Biomass Bioenergy.

[29] Sheth P.N., Babu B.V. (2009). Bioresource Technology Experimental studies on producer gas generation from wood waste in a downdraft biomass gasifier. Bioresour Technol.

[30] Gai C., Dong Y. (2012). Experimental study on non-woody biomass gasification in a downdraft gasifier. Int J Hydrogen Energy.

[31] Sharma A.K. (2009). Experimental study on 75 kW th downdraft (biomass) gasifier system. Renew Energy.

[32] Erlich C., Fransson T.H. (2011). Downdraft gasification of pellets made of wood, palm-oil residues respective bagasse: experimental study. Appl Energy.

[33] Monhol F.A.F., Martins M.F. (2014). Ignition by thermal radiation of polyethylene and human feces combustible wastes: time and temperature to ignition. Adv Mater Res.

[34] Judex J.W., Gaiffi M., Burgbacher H.C. (2012). Gasification of dried sewage sludge: status of the demonstration and the pilot plant. Waste Manage (Oxford).

